# Risk factors for self-harm in prison: a systematic review and meta-analysis

**DOI:** 10.1016/S2215-0366(20)30190-5

**Published:** 2020-08

**Authors:** Louis Favril, Rongqin Yu, Keith Hawton, Seena Fazel

**Affiliations:** a Institute for International Research on Criminal Policy, Faculty of Law and Criminology, Ghent University, Ghent, Belgium; b Centre for Suicide Research, Warneford Hospital, University of Oxford, Oxford, UK; c Department of Psychiatry, Warneford Hospital, University of Oxford, Oxford, UK

## Abstract

**Background:**

Self-harm is a leading cause of morbidity in prisoners. Although a wide range of risk factors for self-harm in prisoners has been identified, the strength and consistency of effect sizes is uncertain. We aimed to synthesise evidence and assess the risk factors associated with self-harm inside prison.

**Methods:**

In this systematic review and meta-analysis, we searched four electronic databases (PubMed, Embase, Web of Science, and PsycINFO) for observational studies on risk factors for self-harm in prisoners published from database inception to Oct 31, 2019, supplemented through correspondence with authors of studies. We included primary studies involving adults sampled from general prison populations who self-harmed in prison and a comparison group without self-harm in prison. We excluded studies with qualitative or ecological designs, those that reported on lifetime measures of self-harm or on selected samples of prisoners, and those with a comparison group that was not appropriate or not based on general prison populations. Data were extracted from the articles and requested from study authors. Our primary outcome was the risk of self-harm for risk factors in prisoners. We pooled effect sizes as odds ratios (OR) using random effects models for each risk factor examined in at least three distinct samples. We assessed study quality on the basis of the Newcastle-Ottawa Scale and examined between-study heterogeneity. The study protocol was registered with PROSPERO, CRD42018087915.

**Findings:**

We identified 35 independent studies from 20 countries comprising a total of 663 735 prisoners, of whom 24 978 (3·8%) had self-harmed in prison. Across the 40 risk factors examined, the strongest associations with self-harm in prison were found for suicide-related antecedents, including current or recent suicidal ideation (OR 13·8, 95% CI 8·6–22·1; *I*^2^=49%), lifetime history of suicidal ideation (8·9, 6·1–13·0; *I*^2^=56%), and previous self-harm (6·6, 5·3–8·3; *I*^2^=55%). Any current psychiatric diagnosis was also strongly associated with self-harm (8·1, 7·0–9·4; *I*^2^=0%), particularly major depression (9·3, 2·9–29·5; *I*^2^=91%) and borderline personality disorder (9·2, 3·7–22·5; *I*^2^=81%). Prison-specific environmental risk factors for self-harm included solitary confinement (5·6, 2·7–11·6; *I*^2^=98%), disciplinary infractions (3·5, 1·2–9·7; *I*^2^=99%), and experiencing sexual or physical victimisation while in prison (3·2, 2·1–4·8; *I*^2^=44%). Sociodemographic (OR range 1·5–2·5) and criminological (1·8–2·3) factors were only modestly associated with self-harm in prison. We did not find clear evidence of publication bias.

**Interpretation:**

The wide range of risk factors across clinical and custody-related domains underscores the need for a comprehensive, prison-wide approach towards preventing self-harm in prison. This approach should incorporate both population and targeted strategies, with multiagency collaboration between the services for mental health, social care, and criminal justice having a key role.

**Funding:**

Wellcome Trust.

## Introduction

More than 11 million individuals are imprisoned worldwide.^1^ People who pass through prisons often have poor health profiles, including high frequencies of self-harm.^2^, ^3^, ^4^ Self-harm is a leading cause of morbidity in prisoners; the annual prevalence of self-harm in prison has been estimated to be 5–6% in men and 20–24% in women,^5^ which greatly exceeds the less than 1% of adults in the general population who self-harm each year.^6^, ^7^, ^8^ Prisoners who self-harm are at a six to eight times increased risk of suicide while incarcerated^9^, ^10^ and remain so after release into the community.^11^, ^12^ Half of people who die by suicide in prison have a recorded history of self-harm,^9^, ^10^ with many deaths occurring within a month of self-harm.^5^ In addition, the impact of self-harm extends to other prisoners^13^ and to prison staff,^14^ and can lead to substantial costs for the prison system, especially if associated with suicide.^15^ Transfer of prisoners to local health-care services for the more severe incidents can further increase costs. Understanding the risk factors for self-harm can help to improve prevention efforts in this population at high risk, particularly if there is evidence of modifiable risk factors.

Previous research has examined a range of individual and environmental correlates of self-harm in prisoners, although findings are inconsistent across primary studies. Reviews^16^, ^17^, ^18^ have been limited by being narrative syntheses of the literature that do not use quantitative methods to evaluate the strength, quality, and consistency of the available evidence. Therefore, we have done a systematic review and meta-analysis of risk factors associated with self-harm inside prison. Our findings could identify appropriate targets for interventions and future treatment trials, and assist decision makers in allocating scarce prison resources.

**Research in context**

**Evidence before this study**

We searched four databases (PubMed, Embase, Web of Science, and PsycINFO) for systematic reviews of self-harm risk factors in adult prisoners published from database inception to Oct 31, 2019. The same keywords were used for each database search for self-harm, “(self-harm* OR suicid* OR attempt* OR NSSI OR self-injur* OR self-mutilat* OR self-destruct* OR poison* OR overdose)”, and prison “(inmate* OR penal OR correction* OR sentence* OR remand OR detainee* OR felon* OR prison* OR incarcerat*)”. No language restrictions were set. We identified three systematic reviews with narrative summaries on risk factors for self-harm, non-suicidal self-injury, and near-lethal suicide attempt. These reviews reported that risk factors span many different individual and environmental domains, although there were many inconsistencies in the magnitude and direction of the effects. We did not identify any reviews that meta-analysed findings or evaluated the strength and consistency of risk factors for self-harm inside prison. We found one meta-analysis published in 2020 that examined the association between childhood maltreatment and suicide attempt in a population who has been in contact with the criminal justice system, but this meta-analysis included non-prisoners, juvenile offenders, and outcomes in the community.

**Added value of this study**

This meta-analysis synthesised data from almost 50 years of research examining risk factors for self-harm in over half a million prisoners. Although we identified many risk factors for self-harm across sociodemographic, criminological, custodial, clinical, and historical domains, the strongest associations were found for suicide-related exposures (suicidal ideation and previous self-harm) and markers of psychiatric morbidity. Modifiable risk factors specific to prison include solitary confinement, disciplinary infractions, physical or sexual victimisation while in custody, and poor social support.

**Implications of all the available evidence**

Our results show that risk factors associated with self-harm in prisoners include a range of potentially modifiable clinical, psychosocial, and environmental factors. These data emphasise the need for a whole-prison approach and multiagency collaboration in the prevention of self-harm.

## Methods

### Search strategy and selection criteria

We did a systematic review and meta-analysis following the Preferred Reporting Items for Systematic Reviews and Meta-analyses guidelines^19^ (known as PRISMA; appendix pp 1–3) in which we searched four electronic databases (PubMed, Embase, Web of Science, and PsycINFO) to identify relevant observational studies on risk factors for self-harm in prisoners published from database inception to Oct 31, 2019. The same keywords were used for each database search for self-harm, “(self-harm* OR suicid* OR attempt* OR NSSI OR self-injur* OR self-mutilat* OR self-destruct* OR poison* OR overdose)”, and prison “(inmate* OR penal OR correction* OR sentence* OR remand OR detainee* OR felon* OR prison* OR incarcerat*)”. No language restrictions were set. Summary estimates were sought.

We supplemented bibliographical database searches by hand-searching the citations and reference lists of relevant articles and previous systematic reviews.^16^, ^17^, ^18^, ^20^ We did targeted searches to identify additional studies by first author names, and contacted experts for unreported or ongoing studies. Through these additional searches, we identified four reports that were not listed in the electronic databases.^21^, ^22^, ^23^, ^24^ We were also able to include new information from two unpublished studies by correspondence with the authors of these studies.^25^, ^26^

LF screened the retrieved references for eligibility. We included primary studies that examined risk factors for self-harm in prison and met the following criteria: the study was cross-sectional, case-control, or cohort in design and included predominantly adult prisoners; the study was based on general prisoner populations (defined as prisoners on remand, sentenced prisoners, or both, sampled from a correctional institution); the study included self-harm within prison as the outcome measure; and the study provided data for an appropriate control or comparison group of unselected prisoners who did not self-harm in prison.

We excluded studies with qualitative or ecological designs, those that reported on lifetime measures of self-harm or outcomes other than self-harm (eg, suicide, suicidal ideation, or a composite measure of suicide risk) or on selected samples of prisoners (eg, sex offenders, prisoners in contact with mental health services, or other groups at high risk of self-harm), and those with a comparison group that was not appropriate (eg, prisoners in hospital wings or prisoners who died by suicide) or not based on general prison populations.

We contacted the authors of studies that did collect information on self-harm in prison but only reported prevalence,^27^ analysed a lifetime history as the outcome variable,^21^, ^28^, ^29^, ^30^ adopted a cluster analytical approach,^31^ or used a subsample of prisoners (those who committed violent acts while incarcerated) as the comparison group.^32^ These seven studies were retained after the required data were obtained from the study authors. The research protocol was registered on PROSPERO (CRD42018087915) before the systematic review was done.

### Data analysis

Data were independently extracted by two researchers (LF and Isabel Yoon). A standardised form was used to extract data and included information on study characteristics (ie, publication year, country, design, and number of prisoners included), sample details (ie, age and sex), outcomes (ie, definition and assessment), and risk factors. Extraction sheets for each study were crosschecked for consistency and any discordance was resolved by discussion between study authors. When the study characteristics were unclear, the corresponding authors of included papers were contacted. When multiple publications from the same population were available, information on risk factors was extracted from the investigation with the largest sample size. Data were only extracted from overlapping publications when a new risk factor was reported.

As the reporting of effect sizes varied between studies, data were converted to a comparable measure for meta-analysis. Odds ratios (ORs) and their 95% CIs were extracted when reported or calculated from available data in the paper (eg, converted from standardised effect sizes) by use of standard formulas.^33^, ^34^ Most studies did not provide adjusted effect sizes and, for many, we had to calculate the ORs on the basis of raw prevalence data. In addition, different studies used contrasting approaches to adjustment (from basic demographics to clinical and custodial factors), which would make adjusted estimates difficult to compare. Therefore, to obtain a consistent measure across studies, data were extracted from the most parsimonious model (ie, the least adjusted model).

LF assessed all studies for risk of bias using the Newcastle-Ottawa Scale for cohort and case-control studies, with 9 points indicating high quality and low risk of bias.^35^ A modification of the Newcastle-Ottawa Scale was adopted for the assessment of cross-sectional studies, which has been used in suicide research before and is out of 8 points.^36^ This scale assesses quality in terms of sample representativeness and size, comparability between respondents and non-respondents, ascertainment of self-harm, and statistical quality. On the basis of these scores, we calculated a summary score (the sum of items divided by the total possible sum) ranging from 0 to 100 and each study was then categorised as low (≤49), moderate (50–74), or high (≥75) quality. Uncertainties were resolved by discussion among study authors.

We grouped risk factors into five categories: sociodemographic, criminological, clinical, custodial, and historical. Three separate outcomes were identified: self-harm, suicide attempt, and non-suicidal self-injury. We have taken a broad definition of self-harm as any act of intentional self-poisoning or self-injury irrespective of the degree of suicidal intent or underlying motive,^5^ which includes both suicide attempt (self-injurious behaviour with inferred or actual intent to die) and non-suicidal self-injury (self-injurious behaviour without any intent to die).^37^ The difficulty in establishing suicidal intent^38^ and the high co-occurrence of both behaviours and their overlapping risk factors^39^ explains our approach of combining non-suicidal self-injury and suicide attempt into a single self-harm outcome. This method is consistent with policy and reporting in many prison jurisdictions, including in England and Wales, which has the largest prison population in western Europe.^1^ In three instances,^21^, ^28^, ^31^ both suicide attempt and non-suicidal self-injury were investigated in the same study sample. To avoid double counting of participants, we contacted the authors from these three studies for data on an aggregated outcome measure of any self-harm: suicide attempt, non-suicidal self-injury, or both. In addition, four studies reported in seven articles^40^, ^41^, ^42^, ^43^, ^44^, ^45^, ^46^ specifically focused on near-lethal suicide attempt, defined as acts that could have been fatal had it not been for intervention or chance, involved methods that are associated with a reasonably high chance of death, or both. We included this outcome as there were no material differences in the effects of risk factors for this outcome compared with other self-harm outcomes. Furthermore, other studies did not differentiate according to the severity or lethality of outcomes and might thus also have included near-lethal self-harm.

To obtain a reliable estimate of pooled effect sizes, analyses were done only on risk factors examined in at least three distinct samples.^33^ Where possible, we examined risk factors for men and women separately. We did the meta-analysis in Stata IC (version 13) using the metan command. For all analyses, we generated random effects models that accounted for the anticipated high heterogeneity between studies resulting from differences in samples, measures, and design. Heterogeneity was estimated by use of the *I*^2^ statistic, which quantifies the percentage of variance across studies that can be attributed to true variation in effect sizes rather than sampling error as low (0–40%), moderate (30–60%), substantial (50–90%), and considerable (75–100%).^47^

The extent to which methodological variations across studies affected the association between risk factors and self-harm was examined by applying meta-regression models (by use of the metareg command). Specifically, univariate meta-regression analyses were done to explore sample size (n<median=0 and n≥median=1) and outcome definition (self-harm=0, suicide attempt=1, and non-suicidal self-injury=2) as possible sources of between-study heterogeneity for all risk factors. The presence of potential publication bias was assessed by examination of asymmetry in funnel plots^48^ and by applying Egger's test^49^ for the top three risk factors that had the most information.

### Role of the funding source

The funder of the study had no role in study design, data collection, data analysis, data interpretation, or writing of the report. LF had full access to the data and all authors had final responsibility for the decision to submit for publication.

## Results

**Figure 1:**
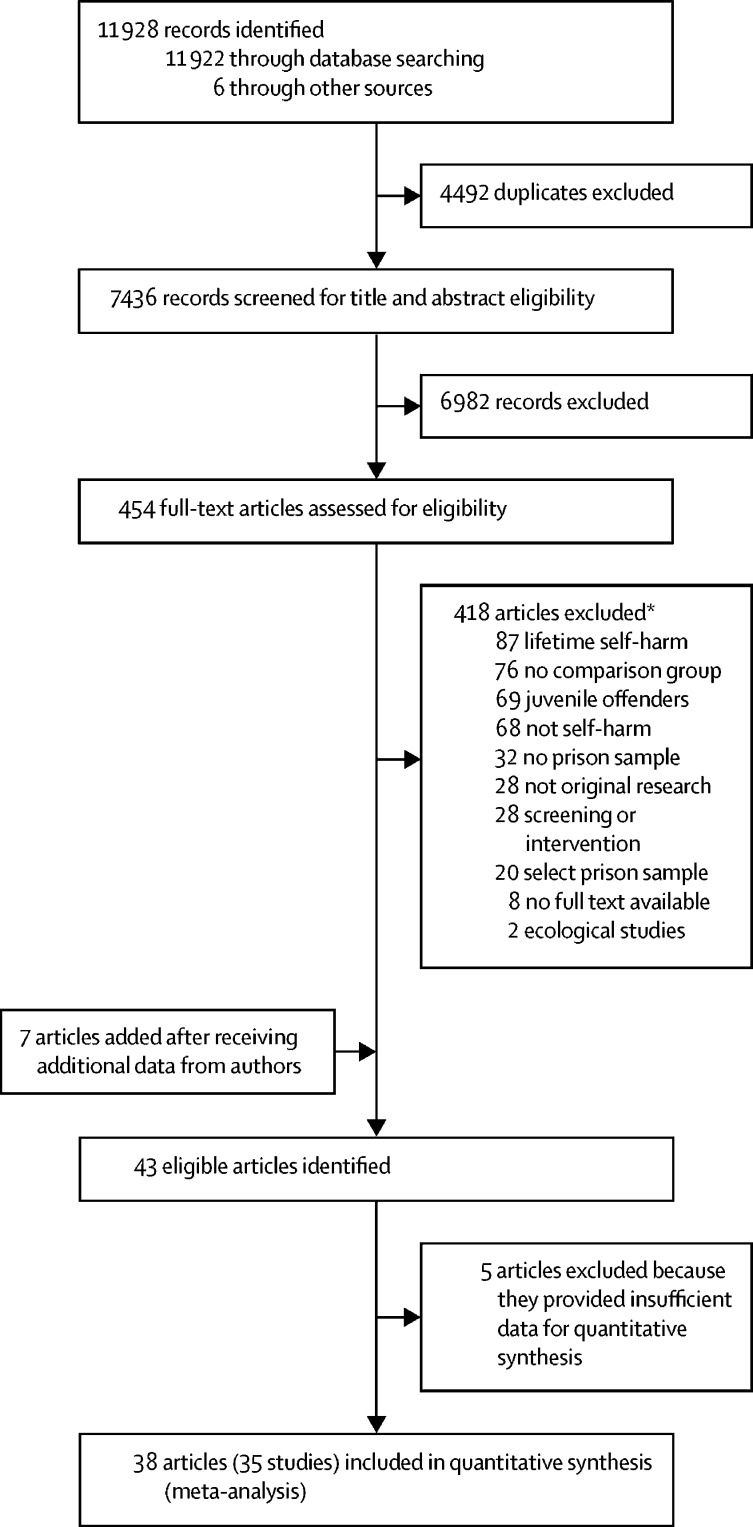
Study selection *Articles were sometimes excluded for multiple reasons; the numbers listed are based on the major reasons for article exclusion.

Our systematic search of the literature identified 7436 unique records for screening, of which 454 (6·1%) full-text reports were examined for eligibility. We included 35 studies reported in 38 articles in the meta-analysis (appendix pp 4–5), comprising a total of 663 735 prisoners, 9·6% of whom were women (figure 1).^5^, ^21^, ^22^, ^23^, ^24^, ^25^, ^26^, ^27^, ^28^, ^29^, ^30^, ^31^, ^32^, ^40^, ^41^, ^42^, ^43^, ^44^, ^45^, ^46^, ^50^, ^51^, ^52^, ^53^, ^54^, ^55^, ^56^, ^57^, ^58^, ^59^, ^60^, ^61^, ^62^, ^63^, ^64^, ^65^, ^66^, ^67^

Included studies were done across 20 countries (11 [31%] in England and Wales) and published from 1972 to 2020. Median sample size was 785 (IQR 142–2119), ranging from 60 to 263 794 prisoners. 20 (57%) of the 35 studies focused solely on either men (k=15) or women (k=5). In 15 studies, the sample included both male and female prisoners, with the mean proportion of women equalling 12·3% (SD 8·7), but only two studies provided data disaggregated by sex.^5^, ^67^ The most frequent designs were case-control studies (k=17; 49%); 12 studies were cross-sectional and six were cohorts. We identified only two prospective studies.^61^, ^65^ The three largest studies were retrospective analyses of routinely collected data,^5^, ^56^, ^67^ accounting for 609 366 (91·8%) of the people in the pooled sample. The most common outcome investigated was self-harm (k=15), followed by (near-lethal) suicide attempt (k=12) and non-suicidal self-injury (k=8). Of all 663 735 prisoners included, 24 978 (3·8%) had self-harmed in prison.

In terms of study quality measured by the Newcastle-Ottawa Scale, of 9 possible points, the median score for the cohort studies was 8·5 (IQR 7–9) and the median score for the case-control studies was 6 (5–8). Of 8 possible points available in the modified Newcastle-Ottawa Scale, the median score for the cross-sectional studies was 6 (5–6). Overall, 18 (51%) of the 35 studies included were judged to be of high quality and four (11%) were categorised as being of low methodological quality (appendix pp 4–5).

There were large variations in the sample sizes contributing to risk estimates. The largest samples were for sex (n=644 812) and violent offending (n=520 581). Only two risk factors (substance use disorder [n=766] and family history of suicide [n=382]) were calculated on the basis of a pooled sample of less than 1000 prisoners. Various sociodemographic factors were associated with self-harm in prison, with pooled ORs ranging from 1·5 to 2·5 (appendix p 6). The three strongest risk factors within this domain were homelessness (OR 2·5, 95% CI 1·8–3·3), unemployment before incarceration (1·6, 1·3–2·1), and being younger than 30 years (2·0, 1·4–2·9). Female sex showed a small increase in risk but a non-significant association with self-harm (1·3, 0·7–2·2). Criminological variables, including violent offences (1·8, 1·3–2·4) and having a previous incarceration (2·0, 1·3–3·1), were risk factors for self-harm in prison (appendix p 7). Being sentenced for more than 5 years (2·3, 1·9–2·7) or serving a life sentence (2·0, 1·2–3·3) doubled the odds of self-harm.

**Figure 2:**
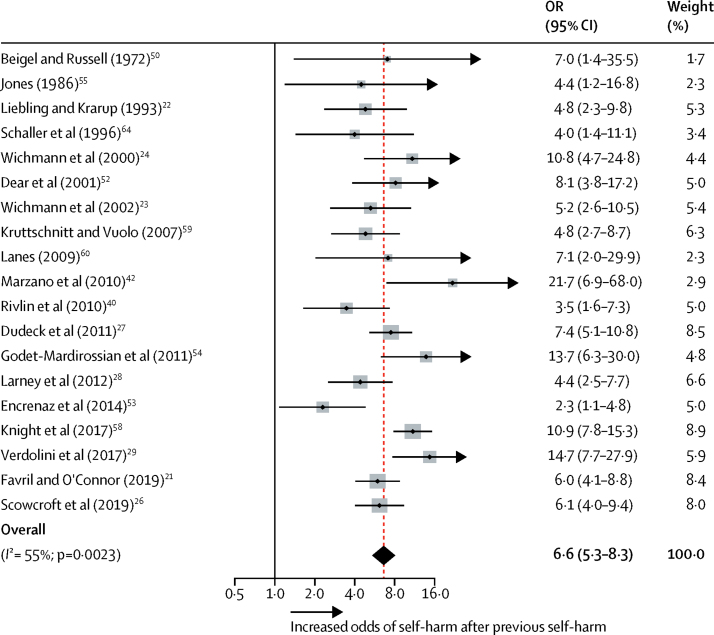
Previous self-harm as a risk factor for self-harm in prison The dots represent the effect sizes and the lines represent the 95% CI from each primary study. The size of the grey boxes reflects the weight attributed to each study. Weights are from random effects analysis. The diamond denotes the pooled summary effect size and CIs. OR=odds ratio.

**Figure 3:**
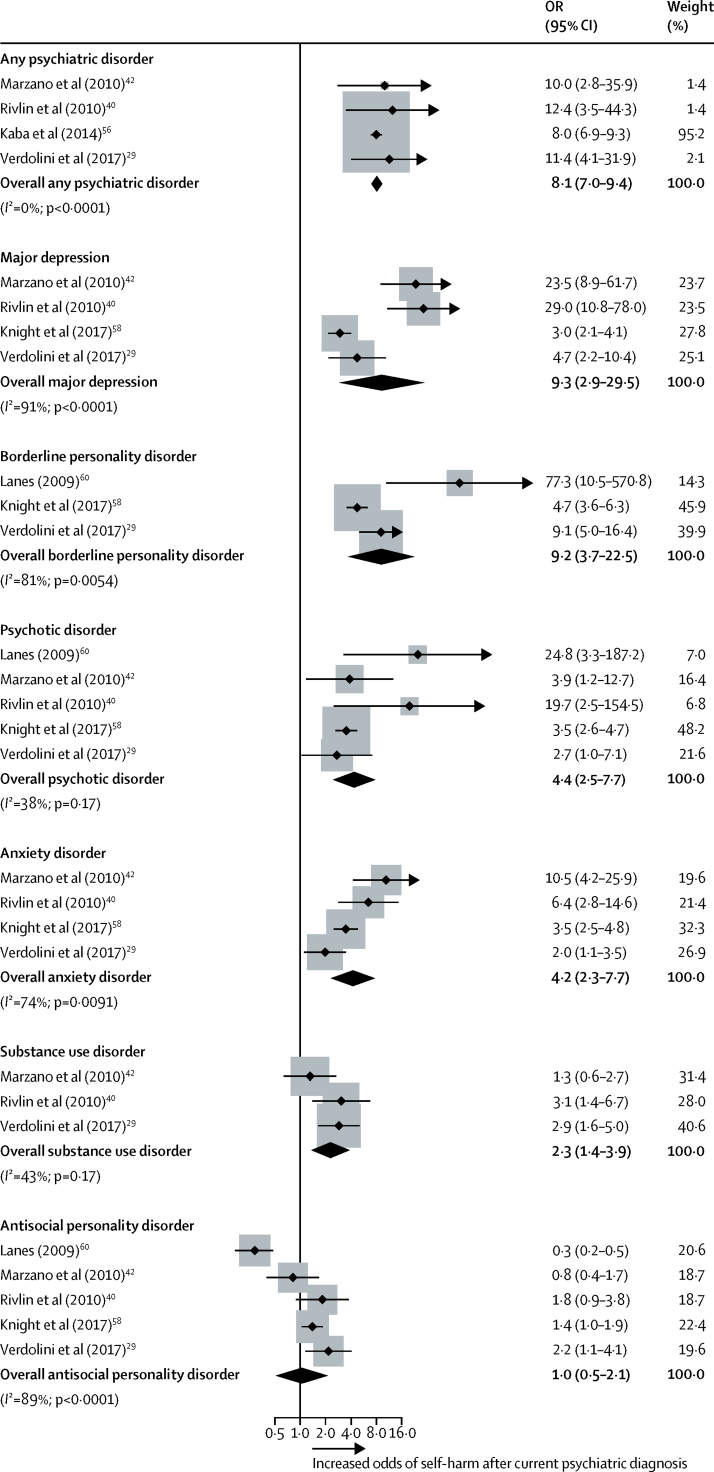
Psychiatric disorders as risk factors for self-harm in prison The dots represent the effect sizes and the lines represent the 95% CI from each primary study. The size of the grey boxes reflects the weight attributed to each study. Weights are from random effects analysis. The diamonds denote the pooled summary effect size and CIs. OR=odds ratio.

Clinical factors showed the strongest associations with self-harm compared with other risk factor domains (OR range 2·0–13·8; table 1). Suicide-related factors, including current or recent (typically within the past month) suicidal ideation, a lifetime history of suicidal ideation, and previous self-harm (table 1, figure 2), had strong effect sizes. Any current psychiatric diagnosis was significantly associated with self-harm, particularly major depression and borderline personality disorder (figure 3). By diagnosis, the odds of self-harm were increased for major depression, borderline personality disorder, psychotic disorder, anxiety disorder, and substance use disorder (OR range 2·3–9·3; table 1). Proxies for psychiatric disorders were also associated with increased odds of self-harm, particularly psychiatric treatment in prison.

**Table 1:** Clinical risk factors for self-harm in prison

		**Studies analysed (k)**	**Participants (n)**	**OR (95% CI)**	***z* score**	**p value**	**Heterogeneity (*I*^2^)**
Suicidal ideation							
	Current or recent	6	7256	13·8 (8·6–22·1)	10·9	<0·0001	49%
	Lifetime history	5	3779	8·9 (6·1–13·0)	11·3	<0·0001	56%
	Overall	11	11 035	10·9 (8·0–14·9)	15·1	<0·0001	58%
Psychiatric treatment							
	In prison	7	30 931	10·5 (4·8–22·8)	5·9	<0·0001	93%
	Before prison	14	11 001	3·7 (2·8–4·9)	9·4	<0·0001	58%
Current psychiatric diagnosis							
	Any psychiatric disorder	4	134 954	8·1 (7·0–9·4)	27·6	<0·0001	0%
	Major depression	4	3908	9·3 (2·9–29·5)	3·8	<0·0001	91%
	Borderline personality disorder	3	3932	9·2 (3·7–22·5)	4·8	<0·0001	81%
	Psychotic disorder	5	4172	4·4 (2·5–7·7)	5·0	<0·0001	38%
	Anxiety disorder	4	3908	4·2 (2·3–7·7)	4·7	<0·0001	74%
	Substance use disorder	3	766	2·3 (1·4–3·9)	3·1	0·0018	43%
	Antisocial personality disorder	5	4172	1·0 (0·5–2·1)	0·1	0·90	89%
Previous self-harm		19	14 154	6·6 (5·3–8·3)	16·3	<0·0001	55%
Impulsivity		5	2153	4·0 (2·6–6·3)	6·2	<0·0001	61%
Hopelessness		4	6201	3·9 (2·1–7·2)	4·3	<0·0001	53%
Current psychotropic medication		8	6400	3·6 (2·5–5·2)	6·6	<0·0001	77%
Psychological distress							
	Severe (cutoff)*	7	9162	3·4 (2·0–5·9)	4·4	<0·0001	85%
	Continuous	6	6345	3·4 (2·1–5·4)	5·1	<0·0001	97%
Physical health problems		9	30 479	2·3 (1·9–2·8)	9·0	<0·0001	14%
History of illicit drug use		10	34 978	2·0 (1·3–3·0)	3·2	0·0015	82%
History of alcohol misuse		8	13 773	1·4 (0·8–2·5)	1·4	0·18	87%

*I*^2^ represents the percentage of variability in estimates of effect size that is attributable to between-study variation (heterogeneity). OR=odds ratio.

* The cutoff value depended on the study and scale.

Several custodial variables were associated with self-harm in prisoners, with ORs ranging from 1·9 to 5·6 (table 2). The strongest associations were found for residing in solitary confinement, having disciplinary infractions, and experiencing physical or sexual victimisation while in prison. We found a non-significant effect for exposure to self-harm in prison; however, a large national study^5^ reported evidence of clustering of self-harm in time and location (OR 1·7, 95% CI 1·5–1·9). All historical life events measured were associated with self-harm (table 3), especially childhood sexual abuse (ie, before the age of 18 years).

**Table 2:** Custody-specific risk factors for self-harm in prison

	**Studies analysed (k)**	**Participants (n)**	**OR (95% CI)**	***z* score**	**p value**	**Heterogeneity (*I*^2^)**
Solitary confinement	11	410 314	5·6 (2·7–11·6)	4·6	<0·0001	98%
Violence or assault perpetration	8	276 968	3·8 (0·9–15·8)	1·8	0·068	99%
Disciplinary infractions	13	302 203	3·5 (1·2–9·7)	2·3	0·019	99%
Sexual or physical victimisation	7	9198	3·2 (2·1–4·8)	5·7	<0·0001	44%
Poor social support	3	2852	3·1 (2·0–4·8)	4·9	<0·0001	62%
Threatened with violence	5	5794	2·6 (2·0–3·3)	7·0	<0·0001	44%
No social contact or visits	5	2153	2·3 (1·5–3·5)	3·9	<0·0001	51%
Not working in prison	3	3311	1·9 (1·5–2·5)	4·8	<0·0001	18%
Single cell accommodation	5	4309	1·5 (0·8–2·9)	1·2	0·23	87%
Exposure to self-harm	4	1708	1·3 (0·5–3·6)	0·6	0·57	89%

*I*^2^ represents the percentage of variability in estimates of effect size that is attributable to between-study variation (heterogeneity). OR=odds ratio.

**Table 3:** Historical risk factors for self-harm in prison

		**Studies analysed (k)**	**Participants (n)**	**OR (95% CI)**	***z* score**	**p value**	**Heterogeneity (*I*^2^)**
Childhood abuse (<18 years of age)							
	Sexual	4	1325	3·9 (2·0–7·5)	4·0	<0·0001	57%
	Physical	3	1183	3·2 (1·4–7·0)	2·9	0·0044	75%
	Emotional	4	3453	3·0 (1·9–4·9)	4·5	<0·0001	71%
	Any abuse	6	9481	2·1 (1·8–2·5)	8·9	<0·0001	0%
Family history of suicide		3	382	3·0 (1·4–6·5)	2·9	0·0041	0%
Sexual abuse ever		5	4985	2·9 (1·9–4·5)	4·7	<0·0001	55%
Local authority care		3	1161	2·4 (1·6–3·5)	4·2	<0·0001	0%
Family history of self-harm		4	1708	1·9 (1·5–2·5)	4·8	<0·0001	0%

*I*^2^ represents the percentage of variability in estimates of effect size that is attributable to between-study variation (heterogeneity). OR=odds ratio.

**Figure 4:**
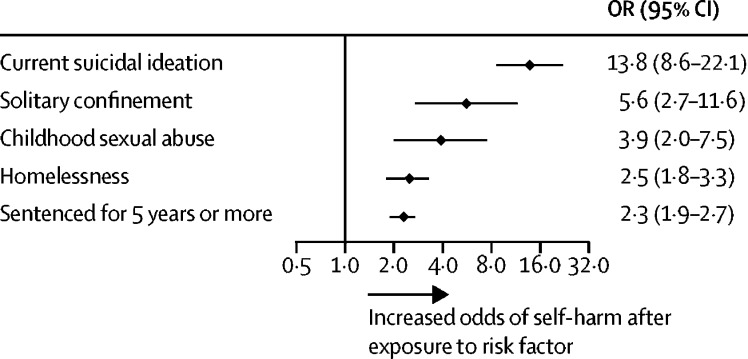
Leading risk factors for self-harm in prison from each domain The dots denote the summary effect sizes from random effects models and the lines denote 95% CIs for all studies. OR=odds ratio.

The leading risk factors from each of the five domains were homelessness, being sentenced for 5 years or more, current suicidal ideation, solitary confinement, and childhood sexual abuse (figure 4). Where possible, we examined risk factors stratified by sex. We identified ten variables that had three or more effect sizes for both men and women (appendix p 8). Pooled estimates for sociodemographic, criminological, and clinical risk factors were largely similar for male (OR range 1·4–6·8) and female (1·6–7·1) prisoners. Whereas some differences were observed in custody-specific risk factors, there was an overall trend for risk estimates to be higher for women than for men, albeit with overlapping confidence intervals (appendix p 8). These findings should, however, be interpreted with caution because we were not able to include data from the 13 studies (n=169 806) that combined sexes. This limitation meant that examining many informative risk factors (eg, those in the historical domain) by sex was not possible.

In meta-regression analyses, we examined sample size and outcome definition as possible sources of between-study heterogeneity in risk estimates (appendix pp 9–11). Sample size was only significantly associated with heterogeneity in meta-regression for nationality (B=1·2; p=0·039) in that studies with larger samples (n≥785) found a larger effect for nationality relative to studies with smaller samples (n<785). Outcome definition was a moderator only for the relationship between major depression and self-harm (B=–2·1; p=0·031), with a stronger effect observed for major depression in studies examining suicide attempt compared with non-suicidal self-injury. Overall, the results suggest that neither sample size, nor outcome definition, explained the heterogeneity in the association between most risk factors and self-harm.

Potential publication bias was examined for three risk factors with the largest number of unique samples. Screening of funnel plots (appendix pp 13–15) suggests that there was no clear publication bias for violent offending (k=24), single marital status (k=20), and previous self-harm (k=19). Similarly, Egger's test was not significant for violent offending (p=0·68), single marital status (p=0·14), and previous self-harm (p=0·53). Additionally, we did post-hoc analyses on the leading risk factors from each domain (ie, homelessness, sentenced for 5 years or more, current suicidal ideation, solitary confinement, and childhood sexual abuse) and again found no evidence for publication bias (all p≥0·18). Sensitivity analyses showed that risk factor estimates did not materially change (both in terms of strength and significance of effects) when low quality studies were excluded from the analyses (appendix p 12).

## Discussion

The present meta-analysis synthesised data from nearly 50 years of research examining risk factors for self-harm in more than half a million prisoners. Across 40 risk factors investigated, the strongest associations with self-harm were past and current suicidality and markers of psychiatric morbidity. Overall, we found strong effects for modifiable clinical and custodial variables, moderate effects for historical variables, and smaller effects for sociodemographic and criminological variables.

Many of the identified risk factors are similar to those found for self-harm in the general population.^37^ Meta-analyses of longitudinal studies have highlighted suicidal ideation, previous self-harm, and psychiatric disorders as replicated risk factors,^68^, ^69^, ^70^, ^71^, ^72^ although the strength of associations was typically stronger in our meta-analysis. However, there was one notable difference. Antisocial personality disorder, despite being strongly associated with self-harm in the community,^73^ was not linked with self-harm in prison.^8^ This difference might reflect the high prevalence of antisocial personality disorder in prisoners, for which diagnostic criteria overlap with the reasons for entering prison.^3^ There was an increased risk of self-harm in female prisoners, although this was not statistically significant. This non-significance contrasts with findings in the general population,^74^ in which female sex as a risk factor for self-harm is stronger, and one high-quality population study of UK prisoners^5^ that reported that the odds of self-harm was four times higher in women than in men. In addition, we found that environmental factors specific to prison, including solitary confinement, disciplinary infractions, victimisation during imprisonment, and poor social support, were clearly associated with self-harm. Although identified as risk factors for suicide in prisoners,^9^, ^10^ we found no clear associations between self-harm and single cell occupancy or remand status. This disparity might reflect differences in risk factors for self-harm as opposed to suicide.^75^ As a whole, this unique pattern of risk factors suggests that a suicide prevention strategy should be tailored to the specificity of the prison setting.

The main clinical implication is the contribution of both individual and environmental risk factors to self-harm in prison. Although we cannot infer causality from this meta-analysis of observational studies, the leading risk factors from each domain suggest that prisoners might import a vulnerability for self-harm into prison (characterised by social disadvantage, trauma, violence, and poor health) that might interact with custody-specific stressors (eg, isolation, victimisation, and long sentences) and thereby increase the likelihood of self-harming in prison. To address both predisposing and precipitating risk factors, the prevention of self-harm in custodial settings might require a comprehensive approach that comprises multilevel interventions, such as screening on reception, staff training (in particular, reducing unhelpful attitudes),^76^ well resourced mental health services, psychosocial treatment, restricting access to lethal means, and multidisciplinary care and support for prisoners at risk.^18^, ^77^ Our systematic review and meta-analysis clearly underscores the evidence for modifiable psychiatric risk factors for self-harm in prisoners, which is consistent with calls for greater health-care involvement in the management and prevention of self-harm in prisons.^3^, ^8^ Universal prison-based strategies that address the identified environmental factors should also be considered, including measures that aim to promote purposeful activity and meaningful social support.^13^, ^18^ This recommendation is supported by evidence on the clustering of self-harm^5^ and suicide^78^ in custody, which suggests that interventions after suicidal behaviour should extend beyond the individual prisoner to others in the same wing or prison who could be at risk. Together, prevention of self-harm will require a comprehensive, prison-wide approach that incorporates both population strategies and targeted strategies, with multiagency collaboration having a key role, including mental health services, social care, and criminal justice agencies.

Strengths of this investigation include a quantitative synthesis, large population numbers, and the inclusion of previously unavailable data. However, there are several limitations. First, despite our rigorous search strategy, which covered four major databases and scanning the reference lists of relevant studies and reviews, adding bibliographical indexes for criminal justice and global health might have identified additional work. Second, the strength of our risk estimates is likely to be overestimated because we did not account for confounding, and risk factors are unlikely to be independent of each other (eg, psychiatric comorbidity). Future work could provide more precise estimates by doing an individual participant meta-analysis, which would allow for the calculation of effect sizes adjusted in the same way. Third, reported associations could be due to reverse causality (eg, solitary confinement as a consequence of self-harm) and prospective studies are necessary to explore whether these risk factors predict self-harm during the course of imprisonment. Fourth, we did not consider the chronicity or frequency of self-harm because most primary studies reported a dichotomous self-harm outcome. Previous work suggests that repetition of self-harm is common, particularly in female prisoners,^5^ and risk factors might differ between a first episode of self-harm in prison and repeat self-harm. Fifth, most of the included cross-sectional studies relied on retrospective self-report of self-harm, which might underestimate the prevalence of self-harm in custody.^79^ This reliance would probably have led to the inflation of our estimates because the more severe end of the self-harm continuum might have been reported. Prospective studies could address this issue, but we only identified two. Sixth, some variables associated with self-harm showed high heterogeneity among risk estimates, so the pooled estimates should be interpreted with caution, and ranges should be also considered. Heterogeneity might be due to national differences in prison regimes and sentencing policies, and diversity in the ethnic compositions of prison populations worldwide. Seventh, by focusing on individual-level determinants of self-harm, we were unable to investigate the role of institutional variables such as prison size, availability of mental health care, and overcrowding. Ecological studies looking at such variables have identified a positive association between overcrowding and self-harm.^80^, ^81^ We are not aware of any studies that have assessed prison size or the availability of mental health care. Finally, we identified no studies from low-income and middle-income countries, and more research in those settings is warranted.

The accurate identification of individuals at risk of self-harm is challenging. Many of the identified risk factors, including suicidal ideation and psychiatric disorders,^3^, ^4^ are unlikely to be predictive because they are common in the mainstream prisoner population. Because prisoners are generally a population at high risk of self-harm, the identification of those at elevated risk of self-harm is a complex task,^16^ and will probably require high-quality methods and external validation.^82^

Given that previous self-harm is among the strongest predictors of future self-harm^68^, ^69^, ^70^ and suicide,^9^, ^10^ effective treatment interventions need development and assessing in prisoners who self-harm. Psychosocial interventions following self-harm,^83^ including forms of cognitive behavioural therapy and group therapy, could be made available in prisons, although current evidence of effectiveness in custodial settings is weak.^84^ Female prisoners are more likely to experience additional stressors around separation from family and children, higher rates of background abusive histories, and bereavement^43^ that might require interventions to be further tailored.

In conclusion, a range of modifiable individual and prison-related factors increase the risk of self-harm in prisoners. Strategies to address these risk factors will potentially require interventions at all levels of the criminal justice system, including diverting people before prison, improvements to mental health care in prison, purposeful activities, and social support, and maintaining these approaches on release. Implementing these interventions will require a multisectorial approach across health, social care, and criminal justice.
