# Supplementary material for: Risk factors for self-harm in prison: a systematic review and meta-analysis
Source: Lancet Psychiatry. 2020 Aug;7(8):682–91. doi: 10.1016/S2215-0366(20)30190-5 (PMC7606912; doi:10.1016/S2215-0366(20)30190-5)

THE LANCET
Psychiatry

Supplementary appendix

This appendix formed part of the original submission and has been peer reviewed. We post it as supplied by the authors.

Supplement to: Favril L, Yu R, Hawton K, Fazel S. Risk factors for self-harm in prison: a systematic review and meta-analysis. *Lancet Psychiatry* 2020; **7:** 682–91.

Table A1. PRISMA checklist.

Section/topic	#	Checklist item	Reported on page #
**TITLE**			
Title	1	Identify the report as a systematic review, meta-analysis, or both.	1
**ABSTRACT**			
Structured summary	2	Provide a structured summary including, as applicable: background; objectives; data sources; study eligibility criteria, participants, and interventions; study appraisal and synthesis methods; results; limitations; conclusions and implications of key findings; systematic review registration number.	1
**INTRODUCTION**			
Rationale	3	Describe the rationale for the review in the context of what is already known.	1
Objectives	4	Provide an explicit statement of questions being addressed with reference to participants, interventions, comparisons, outcomes, and study design (PICOS).	2
**METHODS**			
Protocol and registration	5	Indicate if a review protocol exists, if and where it can be accessed (e.g., Web address), and, if available, provide registration information including registration number.	3
Eligibility criteria	6	Specify study characteristics (e.g., PICOS, length of follow-up) and report characteristics (e.g., years considered, language, publication status) used as criteria for eligibility, giving rationale.	2
Information sources	7	Describe all information sources (e.g., databases with dates of coverage, contact with study authors to identify additional studies) in the search and date last searched.	2
Search	8	Present full electronic search strategy for at least one database, including any limits used, such that it could be repeated.	2
Study selection	9	State the process for selecting studies (i.e., screening, eligibility, included in systematic review, and, if applicable, included in the meta-analysis).	2
Data collection process	10	Describe method of data extraction from reports (e.g., piloted forms, independently, in duplicate) and any processes for obtaining and confirming data from investigators.	3

Data items	11	List and define all variables for which data were sought (e.g., PICOS, funding sources) and any assumptions and simplifications made.	2
Risk of bias in individual studies	12	Describe methods used for assessing risk of bias of individual studies (including specification of whether this was done at the study or outcome level), and how this information is to be used in any data synthesis.	3
Summary measures	13	State the principal summary measures (e.g., risk ratio, difference in means).	3
Synthesis of results	14	Describe the methods of handling data and combining results of studies, if done, including measures of consistency (e.g., I^2) for each meta-analysis.	3–4
Risk of bias across studies	15	Specify any assessment of risk of bias that may affect the cumulative evidence (e.g., publication bias, selective reporting within studies).	5
Additional analyses	16	Describe methods of additional analyses (e.g., sensitivity or subgroup analyses, meta-regression), if done, indicating which were pre-specified.	5
**RESULTS**			
Study selection	17	Give numbers of studies screened, assessed for eligibility, and included in the review, with reasons for exclusions at each stage, ideally with a flow diagram.	5, figure 1
Study characteristics	18	For each study, present characteristics for which data were extracted (e.g., study size, PICOS, follow-up period) and provide the citations.	5, table A2
Risk of bias within studies	19	Present data on risk of bias of each study and, if available, any outcome level assessment (item 12).	5, table A2
Results of individual studies	20	For all outcomes considered (benefits or harms), present, for each study: (a) simple summary data for each intervention group (b) effect estimates and confidence intervals, ideally with a forest plot.	5–7
Synthesis of results	21	Present results of each meta-analysis done, including confidence intervals and measures of consistency.	5–7
Risk of bias across studies	22	Present results of any assessment of risk of bias across studies (item 15).	5
Additional analysis	23	Give results of additional analyses, if done (e.g., sensitivity or subgroup analyses, meta-regression [item 16]).	7

**DISCUSSION**			
Summary of evidence	24	Summarize the main findings including the strength of evidence for each main outcome; consider their relevance to key groups (e.g., healthcare providers, users, and policy makers).	8–9
Limitations	25	Discuss limitations at study and outcome level (e.g., risk of bias), and at review-level (e.g., incomplete retrieval of identified research, reporting bias).	8–9
Conclusions	26	Provide a general interpretation of the results in the context of other evidence, and implications for future research.	8–9
**FUNDING**			
Funding	27	Describe sources of funding for the systematic review and other support (e.g., supply of data); role of funders for the systematic review.	1,5

Table A2. Characteristics of included studies.

First author, year	Country	Design	Mean age (years)	Sample size (% women)	Cases (*n*)	Outcome		Study quality
						Definition	Assessment	
Bani 2019	Italy	Cohort	37·8 (range 18–64)	1406 (0)	95	Self-harm*	Routine data	Moderate
Beigel 1972	United States	Case-control	22·6 (ca), 28·6 (co)	60 (6·7)	30	Suicide attempt	Official records	Low
Cookson 1977	England	Case-control	24 (range 18–64)	78 (100)	39	Self-harm	Official records	Low
Dear 2001	Australia	Case-control	24·7 (ca), 24·9 (co)	142 (9·9)	71	Self-harm	Incident	High
Dudeck 2011	11 European countries	Cross-sectional	39·9	1055 (0)	170	Suicide attempt	Self-report	Low
Encrenaz 2014	France	Cross-sectional	36 (range 18–75)	365 (0)	37	Suicide attempt	Self-report	High
Favril 2019	Belgium	Cross-sectional	37·7 (range 18–77)	1326 (9·3)	197	Self-harm*	Self-report	High
Fleming 2012	Australia	Cross-sectional	37·3 (range 18–73)	146 (37·7)	44	Self-harm	Self-report	Low
Godet-Mardirossian 2011	France	Cross-sectional	37 (range 19–84)	899 (0)	43	Suicide attempt	Self-report	High
Hawton 2014	England and Wales	Case-control	NR	211 354 (6·7)	16 636	Self-harm	Routine data	High
Jones 1986	United States	Case-control	30 (ca), 31 (co)	135 (5·9)	67	NSSI	Official records	Moderate
Kaba 2014	United States	Cohort	NR	134 188 (9·2)	2182	Self-harm	Routine data	High
Kerkhof 1990	Netherlands	Case-control	26·9 (ca), 29·5 (co)	408 (3·4)	198	Suicide attempt	Official records	Moderate
Knight 2017	England and Wales	Cross-sectional	29·0 (range 16–64)	3142 (24·5)	208	NSSI	Self-report	High
Kottler 2018	England	Case-control	29·1 (range 15–62)	5486 (100)	366	Self-harm	Routine data	Moderate
Kruttschnitt 2007	England and United States	Cross-sectional	35·3	2119 (100)	128	Self-harm	Self-report	Moderate
Lanes 2009	United States	Case-control	NR	264 (0)	132	NSSI	Official records	High
Larney 2012	Australia	Cross-sectional	35·5 (range 19–84)	996 (20·0)	89	Self-harm*	Self-report	High
Lekka 2006	Greece	Case-control	33·9	134 (0)	12	Self-harm	Incident	High
Liebling 1993	England	Case-control	Range 16–54	142 (0)	62	Suicide attempt	Incident	Moderate

Martin 2014	Canada	Cohort	34·9	5154 (6·6)	36	NSSI	Routine data	High
Marzano 2010, 2011	England and Wales	Case-control	25·5 (ca), 26·0 (co)	120 (100)	60	Near-lethal suicide attempt	Incident	High
Mohino Justes 2004	Spain	Case-control	19·9 (range 18–25)	95 (0)	19	NSSI	Official records	Moderate
Rivlin 2010, 2013	England and Wales	Case-control	27 (range 18–57)	120 (0)	60	Near-lethal suicide attempt	Incident	High
Sánchez 2018	Spain	Cross-sectional	36·8 (ca), 36·7 (co)	2270 (0)	616	Near-lethal suicide attempt	Self-report	Moderate
Sánchez 2019, 2020	Spain	Cross-sectional	37·2 (range 19–83)	943 (0)	82	Near-lethal suicide attempt	Self-report	Moderate
Schaller 1996	Switzerland	Case-control	NR	341 (16·4)	172	Suicide attempt	Official records	Moderate
Scowcroft 2019	England	Cross-sectional	37·7 (range 18–86)	785 (10·9)	117	Self-harm	Routine data	High
Slade 2014	England	Cohort	32·7	177 (0)	18	Self-harm	Routine data	Moderate
Slade 2019	England	Case-control	35·0 (range 18–83)	965 (0)	175	Self-harm	Routine data	Moderate
Smith 2010	United States	Cohort	31·3 (ca), 34·5 (co)	22 983 (6·8)	189	NSSI	Routine data	High
Verdolini 2017	Italy	Cross-sectional	37·8 (ca), 41·0 (co)	526 (0)	58	NSSI	Self-report	High
Vinokur 2019	Israel	Cohort	NR	263 794 (9·8)	1761	NSSI	Routine data	High
Wichmann 2000	Canada	Case-control	26·0 (ca), 29·9 (co)	1462 (0)	731	Suicide attempt	Official records	Moderate
Wichmann 2002	Canada	Case-control	NR	155 (100)	78	Self-harm	Official records	High

NSSI = non-suicidal self-injury; NR = not reported; ca = cases; co = controls.

* Composite measure of suicide attempt and/or NSSI (data provided by study authors).

Table A3. Sociodemographic risk factors for self-harm in prison.

	k	*n*	OR (95% CI)	*z* score	*p* value	I^2
Homelessness	4	9315	2·5 (1·8–3·3)	6·0	< 0·001	13%
Age						
<30 years	5	213 939	2·0 (1·4–2·9)	3·6	< 0·001	84%
<25 years	6	8113	1·1 (0·5–2·2)	0·2	0·808	87%
Continuous	8	32 367	1·0 (0·9–1·0)	1·6	0·114	74%
Unemployment before prison	8	9454	1·6 (1·3–2·1)	3·9	< 0·001	37%
Financial problems	4	10 686	1·5 (1·3–1·8)	4·9	< 0·001	0%
Low educational attainment	8	9966	1·5 (1·2–1·9)	3·5	0·001	25%
White ethnicity/Caucasian	13	239 186	1·5 (1·2–2·0)	3·0	0·003	78%
Single marital status	20	36 981	1·3 (1·2–1·5)	4·5	< 0·001	32%
Nationality (country of study)	10	277 285	1·4 (0·8–2·2)	1·2	0·215	95%
Female sex	14	644 812	1·3 (0·7–2·2)	0·9	0·377	98%
Having children	9	28 456	1·0 (0·7–1·3)	0·3	0·771	70%

k = number of studies analysed; *n* = total number of participants included in pooled analyses; OR = pooled odds ratio and its 95% confidence interval (CI); I^2 = percentage of variability in effect size estimates that is attributable to between-study variation (heterogeneity).

Table A4. Criminological risk factors for self-harm in prison.

	k	*n*	OR (95% CI)	*z* score	*p* value	I^2
Sentence length*						
<1 year	8	217 903	0·7 (0·5–1·2)	1·3	0·190	87%
<4 years	4	213 864	1·0 (0·7–1·4)	0·2	0·813	86%
5+ years	4	5093	2·3 (1·9–2·7)	8·6	< 0·001	0%
Life sentence	5	214 382	2·0 (1·2–3·3)	2·6	0·009	83%
Prior incarceration	16	300 759	2·0 (1·3–3·1)	3·0	0·003	97%
Violent offending	24	520 581	1·8 (1·3–2·4)	3·6	< 0·001	96%
Remand status	16	224 218	1·0 (0·7–1·6)	0·1	0·915	96%

k = number of studies analysed; *n* = total number of participants included in pooled analyses; OR = pooled odds ratio and its 95% confidence interval (CI); I^2 = percentage of variability in effect size estimates that is attributable to between-study variation (heterogeneity). * Sentenced prisoners only.

Table A5. Risk factors for self-harm in prison, by sex.

	MEN					WOMEN				
	k	OR (95% CI)	*z* score	*p*	I^2	*k*	OR (95% CI)	*z* score	*p*	I^2
White ethnicity	7	1·6 (1·1–2·3)	2·6	0·009	78%	4	1·5 (0·7-3·1)	1·1	0·288	87%
Single marital status	10	1·4 (1·2–1·6)	3·7	< 0·001	38%	3	1·6 (1·1-2·4)	2·5	0·013	0%
Violent offending	12	1·4 (1·2–1·7)	3·6	< 0·001	80%	6	2·4 (1·8–3·2)	5·8	< 0·001	45%
Remand status	10	1·1 (0·6–2·0)	0·4	0·704	95%	3	1·9 (0·6–6·6)	1·1	0·288	89%
Prior incarceration	8	1·5 (1·1–2·1)	2·3	0·023	75%	3	3·2 (2·6–3·9)	11·5	< 0·001	0%
Prior self-harm	8	6·8 (4·3–10·6)	8·4	< 0·001	68%	3	7·1 (3·4–15·0)	5·1	< 0·001	64%
Psychiatric treatment	5	5·2 (3·0–9·1)	5·8	< 0·001	42%	3	4·7 (1·4–16·2)	2·5	0·014	84%
Violence/assault	4	1·9 (0·9–3·8)	1·7	0·083	98%	3	7·1 (2·7–18·8)	4·0	< 0·001	89%
Disciplinary infraction	8	2·5 (1·8–3·4)	5·7	< 0·001	79%	3	3·8 (0·8–18·4)	1·6	0·103	96%
Solitary confinement	6	2·9 (1·6–5·4)	3·4	0·001	94%	3	4·2 (0·8–22·3)	1·7	0·094	87%

k = number of studies analysed; OR = pooled odds ratio and its 95% confidence interval (CI); I^2 = percentage of variability in effect size estimates that is attributable to between-study variation (heterogeneity).

Table A6. Univariate meta-regression analyses assessing sample size and outcome definition as possible sources of heterogeneity for each risk factor.

	SAMPLE SIZE			OUTCOME DEFINITION		
	B	SE(B)	*p*	B	SE(B)	*p*
Female sex	0·171	0·379	0·660	–0·126	0·178	0·493
Age <30 years	0·105	0·515	0·851	0·309	0·309	0·268
Age <25 years	–0·570	0·766	0·498	–0·234	0·590	0·713
Age (continuous)	–0·210	0·221	0·378	–0·021	0·128	0·876
White ethnicity/Caucasian	–0·248	0·336	0·477	0·205	0·187	0·297
Nationality (country of study)	1·209	0·489	0·039	–0·082	0·393	0·840
Single marital status	0·214	0·136	0·133	–0·017	0·112	0·878
Low educational attainment	–0·178	0·269	0·533	0·432	0·353	0·267
Unemployment before prison	0·405	0·170	0·055	0·722	0·567	0·250
Having children	–0·469	0·297	0·158	–0·226	0·236	0·370
Homelessness	–0·049	0·535	0·936	–0·105	0·455	0·839
Financial problems	0·082	0·431	0·866	0·235	0·175	0·311
Violent offending	0·215	0·262	0·421	0·008	0·169	0·962
Remand status	–0·666	0·383	0·104	0·500	0·265	0·080
Prior incarceration	–0·091	0·398	0·823	–0·033	0·264	0·904
Sentence length <1 year	0·558	0·640	0·417	0·270	0·651	0·693
Sentence length <4 years	0·307	0·529	0·620	–0·441	0·194	0·151
Sentence length 5+ years	—[a]	—[a]	—[a]	0·018	0·203	0·938
Life sentence	0·489	0·566	0·451	0·319	0·520	0·583

Current/recent suicidal ideation	–0·148	0·564	0·807	–0·126	0·400	0·768
History of suicidal ideation	0·035	0·470	0·946	–0·106	0·273	0·723
Prior self-harm	0·197	0·244	0·431	0·284	0·143	0·063
Any psychiatric diagnosis	–0·344	0·355	0·434	0·216	0·234	0·453
Major depression	–1·577	1·089	0·285	–2·110	0·383	0·031
Substance use disorder	—[b]	—[b]	—[b]	0·378	0·692	0·682
Anxiety disorder	–0·332	0·958	0·762	–1·059	0·439	0·137
Psychotic disorder	–0·561	0·932	0·590	–0·481	0·744	0·564
Antisocial personality disorder	0·367	1·011	0·741	–0·247	0·857	0·792
Borderline personality disorder	–1·500	1·688	0·538	—[c]	—[c]	—[c]
History of alcohol abuse	0·298	0·644	0·660	0·387	0·370	0·336
History of illicit drug abuse	0·032	0·454	0·945	–0·235	0·264	0·399
Psychotropic medication	–0·557	0·514	0·320	0·020	0·413	0·962
Psychiatric treatment (prior)	–0·205	0·341	0·559	0·247	0·174	0·180
Psychiatric treatment (prison)	0·196	0·777	0·811	0·566	0·528	0·333
Severe distress (cut-off)	–0·892	0·706	0·262	0·464	0·552	0·439
Distress (continuous)	–1·026	0·902	0·307	0·667	1·072	0·561
Hopelessness	–1·034	0·414	0·130	0·265	0·451	0·616
Impulsivity	0·521	0·557	0·418	–0·190	0·563	0·758
Physical health problems	–0·042	0·232	0·861	0·160	0·116	0·210
Victim of abuse	–0·165	0·537	0·771	–0·010	0·317	0·976
Threatened with violence	0·204	0·321	0·571	0·369	0·152	0·093

No social contact or visits	0·558	0·412	0·269	–0·558	0·412	0·269
Poor social support	–0·277	0·606	0·727	0·277	0·606	0·727
Violence/assault perpetration	0·914	1·034	0·411	–0·480	0·541	0·409
Disciplinary infractions	0·587	0·647	0·383	0·375	0·395	0·363
Solitary confinement	–0·291	0·639	0·660	0·048	0·394	0·905
Exposure to self-harm	1·376	0·559	0·133	–1·376	0·559	0·133
Not working in prison	–0·510	0·369	0·399	—[d]	—[d]	—[d]
Single cell accommodation	0·075	1·153	0·952	–0·484	0·813	0·593
Childhood sexual abuse	–0·469	0·925	0·663	—[d]	—[d]	—[d]
Childhood emotional abuse	–0·581	0·793	0·540	—[d]	—[d]	—[d]
Childhood physical abuse	–0·894	0·695	0·421	—[d]	—[d]	—[d]
Any childhood abuse	–0·237	0·303	0·478	0·122	0·330	0·729
Sexual abuse ever	0·403	0·463	0·448	0·315	0·244	0·287
Local authority care	0·225	0·470	0·716	—[d]	—[d]	—[d]
Family history of suicide	—[b]	—[b]	—[b]	—[d]	—[d]	—[d]
Family history of self-harm	0·105	0·308	0·766	–0·105	0·308	0·766

Sample size: based on the median sample size as the cut-off value for smaller ($n < 785$) and larger ($n \geq 785$) studies.

Outcome definition: self-harm = 0, suicide attempt = 1, NSSI = 2.

[a] Meta-regression analysis was not possible because the pooled effect size was based only on studies with samples $n \geq 785$.

[b] Meta-regression analysis was not possible because the pooled effect size was based only on studies with samples $n < 785$.

[c] Meta-regression analysis was not possible because the pooled effect size was based only on a single self-harm outcome (NSSI).

[d] Meta-regression analysis was not possible because the pooled effect size was based only on a single self-harm outcome (suicide attempt).

Table A7. Risk factors for self-harm in prison when excluding low-quality studies.

	k	*n*	OR (95% CI)	*z* score	*p* value	I^2
Female sex	12	644 606	1·3 (0·7–2·3)	0·9	0·360	98%
Age <30 years	3	213 715	2·0 (1·3–3·2)	3·2	0·001	91%
Age (continuous)	7	31 312	1·0 (0·9–1·0)	1·5	0·124	78%
White ethnicity/Caucasian	12	239 126	1·6 (1·2–2·1)	3·4	0·001	77%
Single marital status	17	35 720	1·3 (1·2–1·5)	4·0	< 0·001	41%
Having children	7	27 255	1·0 (0·7–1·6)	0·1	0·937	78%
Violent offending	20	519 243	1·9 (1·3–2·6)	3·6	< 0·001	97%
Remand status	15	224 140	1·1 (0·7–1·7)	0·3	0·756	96%
Prior incarceration	14	299 644	1·8 (1·1–3·1)	2·2	0·031	97%
History of suicidal ideation	4	3633	8·7 (5·6–13·3)	9·8	< 0·001	66%
Prior self-harm	16	13 039	6·5 (5·1–8·5)	14·3	< 0·001	60%
History of illicit drug abuse	9	34 832	1·9 (1·2–2·9)	2·9	0·004	83%
Psychotropic medication	7	6254	3·8 (2·5–5·8)	6·4	< 0·001	80%
Psychiatric treatment (prior)	13	10 855	3·6 (2·7–4·8)	8·9	< 0·001	60%
Severe distress (cut-off)	5	7961	5·1 (2·7–9·6)	5·0	< 0·001	82%
Distress (continuous)	6	5290	2·5 (1·9–3·3)	6·4	< 0·001	97%
Sexual abuse ever	4	3930	3·3 (2·0–5·3)	4·9	< 0·001	35%

k = number of studies analysed; *n* = total number of participants included in pooled analyses; OR = pooled odds ratio and its 95% confidence interval (CI); I^2 = percentage of variability in effect size estimates that is attributable to between-study variation (heterogeneity).
Risk estimates for the variables not presented in this table were based only on studies of moderate and/or high quality.

Figure A1. Funnel plot for violent offending (*k* = 24).

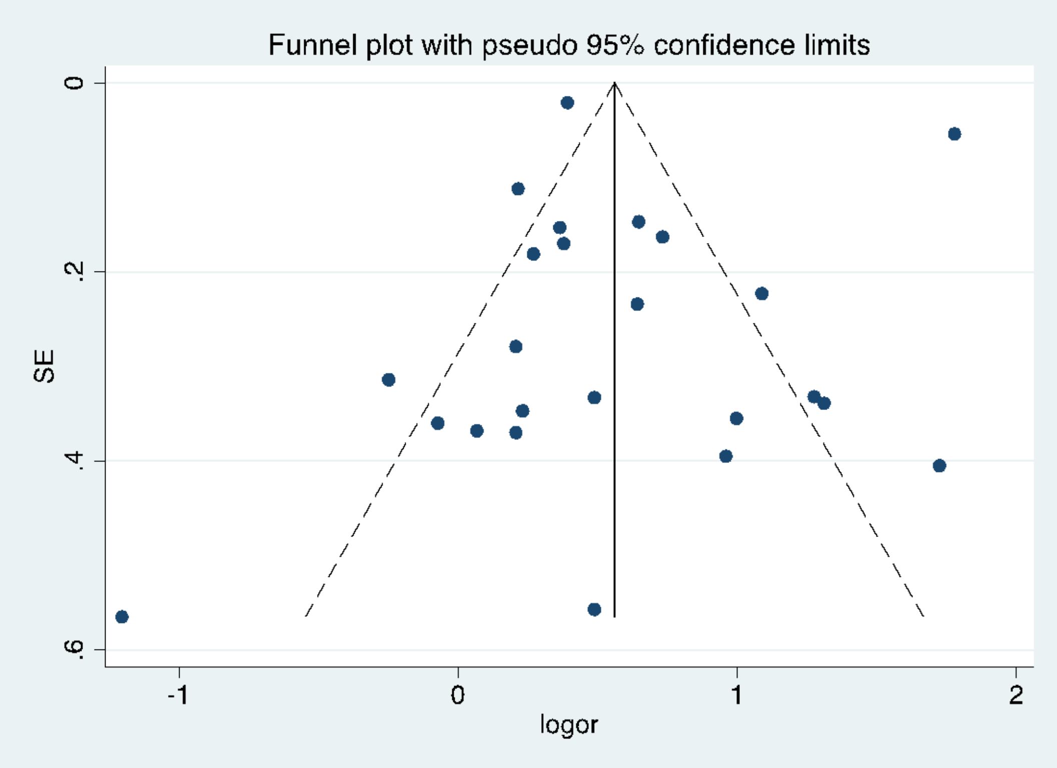

Figure A2. Funnel plot for single status (*k* = 20).

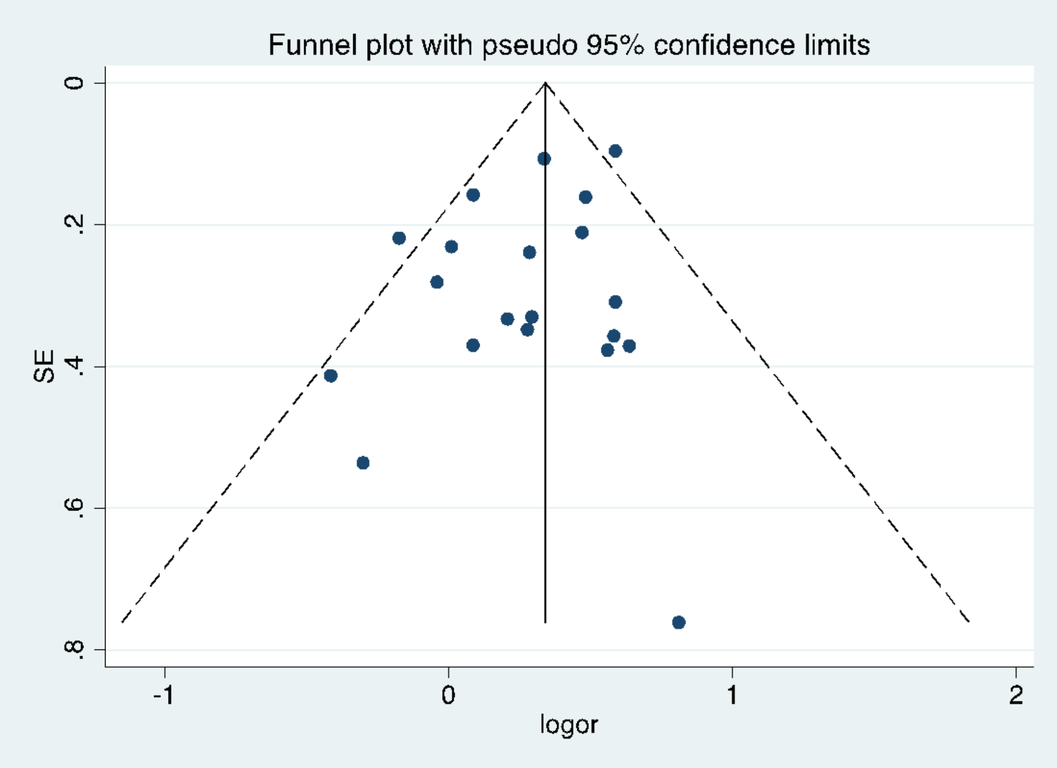

Figure A3. Funnel plot for prior self-harm (*k* = 19).

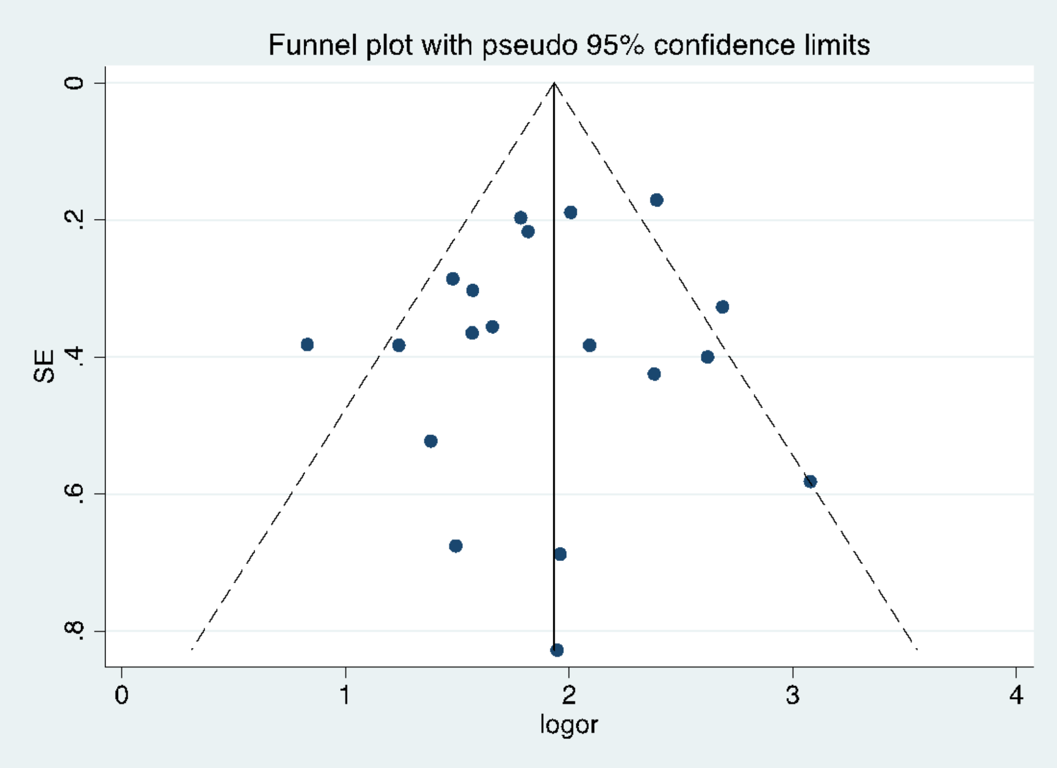

Supplement: Supplementary appendix [file mmc1.pdf]
